# Agouti protein, mahogunin, and attractin in pheomelanogenesis and melanoblast-like alteration of melanocytes: a cAMP-independent pathway

**DOI:** 10.1111/j.1755-148X.2009.00582.x

**Published:** 2009-10

**Authors:** Tokimasa Hida, Kazumasa Wakamatsu, Elena V Sviderskaya, Andrew J Donkin, Lluis Montoliu, M Lynn Lamoreux, Bin Yu, Glenn L Millhauser, Shosuke Ito, Gregory S Barsh, Kowichi Jimbow, Dorothy C Bennett

**Affiliations:** 1 Division of Basic Medical Sciences, St. George’s, University of LondonCranmer Terrace, London, UK; 2 Department of Dermatology, Sapporo Medical UniversitySapporo, Hokkaido, Japan; 3 Department of Chemistry, Fujita Health University School of Health, SciencesToyoake, Aichi, Japan; 4 Department of Molecular and Cellular Biology, Centro Nacional de Biotecnología (CNB-CSIC), Campus de Cantoblanco, and Centro de Investigación Biomédica en Red de Enfermedades Raras (CIBERER)ISCIII, Madrid, Spain; 5 Comparative Genetics Program, Texas A&M University, College StationTX, USA; 6 Department of Chemistry and Biochemistry, University of CaliforniaSanta Cruz, CA, USA; 7 Howard Hughes Medical Institute and the Department of Pediatrics and Genetics, Stanford University Medical CenterStanford, CA, USA

**Keywords:** pheomelanin, agouti, Mc1r, cAMP, melanoblast, attractin, mahogunin

## Abstract

Melanocortin-1 receptor (MC1R) and its ligands, α-melanocyte stimulating hormone (αMSH) and agouti signaling protein (ASIP), regulate switching between eumelanin and pheomelanin synthesis in melanocytes. Here we investigated biological effects and signaling pathways of ASIP. Melan-a non agouti (*a/a*) mouse melanocytes produce mainly eumelanin, but ASIP combined with phenylthiourea and extra cysteine could induce over 200-fold increases in the pheomelanin to eumelanin ratio, and a tan-yellow color in pelletted cells. Moreover, ASIP-treated cells showed reduced proliferation and a melanoblast-like appearance, seen also in melanocyte lines from yellow (*A*^*y*^*/a* and *Mc1r*^*e*^*/ Mc1r*^*e*^) mice. However ASIP-YY, a C-terminal fragment of ASIP, induced neither biological nor pigmentary changes. As, like ASIP, ASIP-YY inhibited the cAMP rise induced by αMSH analog NDP-MSH, and reduced cAMP level without added MSH, the morphological changes and depigmentation seemed independent of cAMP signaling. Melanocytes genetically null for ASIP mediators attractin or mahogunin (*Atrn*^*mg-3J/mg-3J*^ or *Mgrn1*^*md-nc/md-nc*^) also responded to both ASIP and ASIP-YY in cAMP level, while only ASIP altered their proliferation and (in part) shape. Thus, ASIP–MC1R signaling includes a cAMP-independent pathway through attractin and mahogunin, while the known cAMP-dependent component requires neither attractin nor mahogunin.

**Significance**

Many animal colors and patterns as well as human hair and eye color are genetically determined through the relative amounts of eumelanin and pheomelanin synthesized. Humans with red (pheomelanic) hair in general, and with specific hypomorphic *MC1R* mutations in particular, have increased susceptibility to melanoma, and the mechanism for this remains uncertain. Melanin, especially pheomelanin, has been implicated in mutagenesis in melanocytes by ultraviolet light, particularly UVA, via release of reactive oxygen species that can damage DNA. Ability to regulate the switch between eumelanin and pheomelanin synthesis in cultured melanocytes would greatly aid studies of mutagenesis and photocarcinogenesis in these cells. This work may also shed some light on the longstanding puzzle of how cAMP levels and ASIP can control both melanocytic differentiation and pigment-type switching.

## Introduction

The diverse patterns of mammalian coat color are determined by the quantity and distribution of just two types of organic pigment: eumelanin (black to brown) and pheomelanin (yellow to red) (Barsh, 2006; Ito, 2003). Both are produced by melanocytes in the hair bulbs and basal epidermis. They are synthesized and accumulate in melanosomes, specialized organelles which are transferred to keratinocytes of the hair and epidermis (Jimbow et al., 2000). Each melanocyte can produce either eumelanin or pheomelanin, depending on the hormonal and chemical environment (Barsh, 2006). In agouti (wild-type) mice the dorsal hairs are black with a sub-apical yellow band because, in the hair cycle, follicular melanocytes produce first eumelanin, then pheomelanin, then eumelanin again (pigment-type switching) (Barsh, 2006).

Genetic studies of coat color in mice have contributed greatly to our understanding of the mechanisms of melanin synthesis and its regulation (Bennett and Lamoreux, 2003). Two major loci are central to pigment-type switching in mouse. One is the agouti locus encoding agouti signal protein (ASIP), with mutants including non agouti (*a*, giving a eumelanic black mouse in the absence of other mutations) and dominant yellow (*A*^*y*^); the other is the melanocortin-1 receptor (*Mc1r*) locus, formerly extension (*e*), also with both eumelanic and pheomelanic mutants (e.g. recessive yellow*, Mc1r*^*e*^) (Barsh, 2006). Melanocortin-1 receptor (MC1R) is a cell-surface G-protein-coupled receptor for which the best-known agonist is the soluble peptide αMSH, cleaved from the precursor pro-opiomelanocortin (POMC) in the pituitary and skin (Barsh, 2006; García-Borrón et al., 2005). Binding of αMSH to MC1R is known to activate adenylate cyclase and cAMP synthesis, promoting eumelanin synthesis through both post-translational [review: (Bennett, 1989)] and transcriptional pathways via microphthalmia-related transcription factor (MITF) (Bertolotto et al., 1998; Levy et al., 2006). Microphthalmia-related transcription factor is a master regulator for eumelanogenesis, melanocyte differentiation, proliferation, and survival. It promotes transcription of melanocyte-specific gene products including melanosomal enzymes tyrosinase, TYRP1 and DCT and the matrix protein SILV/PMEL (Levy et al., 2006). Synthesis of both eumelanin and pheomelanin starts from tyrosine oxidation catalyzed by tyrosinase (Ito, 2003). The resulting dopaquinone can be a precursor for either eumelanin synthesis, promoted by TYRP1 and DCT, or pheomelanin in the presence of high cysteine concentrations and/or low tyrosinase activity (Ito, 2003; Land et al., 2003).

Agouti signaling protein is a soluble protein of 131 amino acids, apparently secreted by dermal papilla cells in hair bulbs. It competitively antagonizes αMSH at the MC1R and inhibits the eumelanogenic signal, downregulating melanogenic enzymes and leading to pheomelanin synthesis (Barsh, 2006; Millar et al., 1995).

The above account does not however explain certain data on pigment-type switching. Firstly, a homozygous null mutation of *Pomc* has no effect on the black pigmentation of *a/a* mice (Slominski et al., 2005), and has also been reported in a black-haired human (Clément et al., 2008) consistent with reports that MC1R has constitutive activity (Barsh, 2006; García-Borrón et al., 2005; Sanchez-Mas et al., 2004). While ASIP requires the MC1R to affect melanocytes, it can signal in the absence of added αMSH (Abdel-Malek et al., 2001; Sakai et al., 1997; Sviderskaya et al., 2001), thus acting as an inverse agonist at the MC1R rather than just an MSH antagonist (Barsh, 2006). Secondly, molecules outside the cAMP pathway are indispensable for ASIP to signal pheomelanogenesis. Mice with null mutations at the attractin (*Atrn,* formerly mahogany) or mahogunin (*Mgrn1,* formerly mahoganoid) loci produce eumelanin and no pheomelanin, even in the presence of ASIP (He et al., 2001, 2003). Attractin is a type I transmembrane protein proposed to be an obligatory accessory receptor for ASIP, while MGRN1 is an E3 ubiquitin ligase (Barsh, 2006). Although their precise contributions to ASIP signaling remain unclear, ATRN and MGRN1 are components with ASIP and MC1R of a conserved biochemical and genetic pathway (He et al., 2003). Thirdly, ASIP has not been found to enhance pheomelanogenesis in cultured melanocytes, although ASIP can inhibit cAMP generation in MC1R-expressing cells including melanocytes (Abdel-Malek et al., 2001; Le Pape et al., 2008; Ollmann et al., 1998). No culture conditions have been developed in which melanocytes produce exclusively pheomelanin, even with ASIP. This suggests that either a factor(s) present in vivo is missing, or that something present in cultures may prevent pheomelanogenesis.

To aid understanding of ASIP signaling, we have sought to develop culture conditions under which ASIP contributes to overt pheomelanin synthesis by the melan-a immortal murine melanocyte line. We report conditions enabling substantially increased pheomelanin/eumelanin ratios and visibly yellow cell pellets. To elucidate ASIP signaling, we also established new mouse melanocyte lines of four relevant mutant genotypes, namely *A*^*y*^ mutant cells from mice with constitutive ASIP synthesis from a housekeeping promoter, *Mc1r*^*e*^*/ Mc1r*^*e*^ cells with a non-functional MC1R receptor, and melanocytes with null mutations of *Atrn* and *Mgrn1*. Taken together, the data demonstrate that ASIP signaling can reduce melanocyte growth and induce morphological dedifferentiation as well as affecting pigmentation. These biological effects are mimicked in genetically yellow melanocytes without added ASIP, are incomplete in *Atrn-* and *Mgrn1-*null melanocytes, appear independent of cAMP downregulation, and require the amino terminus of ASIP.

## Results

### Melanoblast-like morphology and reduced proliferation of melanocytes grown with ASIP

**Figure 1:**
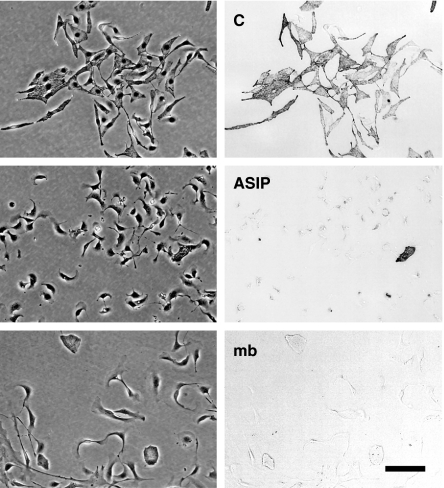
Morphological and pigmentary changes induced in melan-a cells by agouti signaling protein (ASIP). Paired phase contrast (left) and bright-field (right, to show eumelanin) images of melan-a cells grown in normal growth medium (C, control); or in the same medium with 10 nM ASIP added for the last 5 days (ASIP). No phenylthiourea (PTU) or extra cysteine was present (see text). (mb): normal murine melanoblasts in a primary neonatal epidermal melanocyte culture, 7 days after explantation, showing similarity to melan-a cells grown with ASIP. Bar = 100 μm.

Melan-a cells (*a/a*) grown with 12-*O*-tetradecanoyl phorbol-13-acetate (TPA) normally show a dendritic, well-differentiated appearance with abundant eumelanosomes (Figure 1) (Bennett et al., 1987). The appearance of these cells was dramatically changed however within 5 days’ culture with 10 nM murine ASIP. The cells became shorter, smaller, and less dendritic, often with a crescent shape and ruffled membrane on the convex side, and markedly less pigmented (Figure 1). This appearance resembled that of normal and immortal melanoblasts – unpigmented melanocyte precursors – in similar media (Figure 1; Sviderskaya et al., 1995). Melanoblasts are the predominant growing population in primary neonatal epidermal cultures in our melanocyte medium in the first 7–10 days, after which they differentiate into melanocytes (Sviderskaya et al., 1995). Agouti signaling protein was apparently inducing rapid morphological dedifferentiation of melan-a cells, although a few cells (possibly non-dividing) remained well-pigmented. Transitional forms were rare (Figure 1). The proliferation rate of melan-a cells was also significantly reduced in the presence of ASIP (data in a later section), as also reported by Le Pape et al. (2008).

To assess the specificity of these morphological changes, the effect of ASIP was tested on melan-e2 melanocytes, derived from *Mc1r*^*e*^*/ Mc1r*^*e*^ mice lacking the normal MC1R. Agouti signaling protein had no detectable effect on these cells, indicating that the morphological effect is specific (details in a later section).

### Pheomelanin/eumelanin ratio markedly enhanced by ASIP, cysteine, and PTU

**Figure 2:**
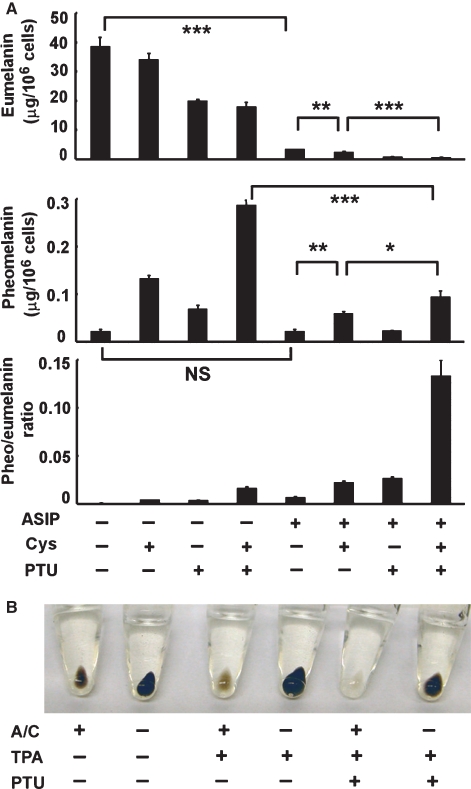
Marked increases in pheomelanin/eumelanin ratio and tan coloration by agouti signaling protein (ASIP), cysteine and phenylthiourea (PTU). Cells were subcultured as needed to prevent confluency. (A) Eumelanin and pheomelanin content of melan-a cells after 14 days’ growth with 300 μM PTU followed by 14 days’ growth in standard medium [containing 12-*O*-tetradecanoyl phorbol-13-acetate (TPA)] with the indicated additives. Data show mean ± SEM of three different samples. (ASIP): 10 nM ASIP; (Cys): 2 mM cysteine plus 100 μM mercaptoethanol; (PTU): 100 μM PTU. (B) Pellets of cells after 14 days’ growth with 500 μM PTU followed by 5 days’ growth with the indicated additives. (A/C): 10 nM ASIP, 2 mM cysteine and 100 μM mercaptoethanol; (TPA): 200 nM TPA; (PTU): PTU at 500 μM for 2 days and 100 μM for 3 days. Significances of selected differences by Student’s *t*-test (two-tailed) are shown: *P < 0.05; **P < 0.01; ***P < 0.001, (NS): P > 0.05, not significant.

Pheomelanin and eumelanin content and their ratio in melan-a cells were quantitated by chemical degradation and high performance liquid chromatography. In preliminary experiments, no increase of pheomelanin content was detected on growth with ASIP alone, as previously reported by others. Therefore other relevant factors, the cysteine concentration and tyrosinase activity [using the tyrosinase inhibitor phenylthiourea (PTU)], were modulated together with ASIP addition. Cells were preincubated with PTU for 2 weeks beforehand to allow dilution of preexisting eumelanin by cell proliferation (melan-a cells in the absence of keratinocytes do not significantly secrete or degrade melanin, as recently confirmed by Le Pape et al. (2008)), and monitoring only of melanin synthesized during the experiment. In a typical experiment, eumelanin content was reduced around 10-fold after growth with 10 nM ASIP for 14 days, while pheomelanin content was not affected (Figure 2). The pheomelanin/eumelanin ratio was thus increased over 10-fold. In the presence of ASIP, an additional 2 mM cysteine reduced eumelanin content further, and increased pheomelanin content, while continuation of PTU addition (100 μM) substantially reduced eumelanin content without significantly affecting pheomelanin (Figure 2). With ASIP, cysteine, and PTU together pheomelanin content was around four times that with ASIP alone. In the experiment shown, the pheomelanin and eumelanin ratio in cells grown with ASIP, cysteine, and PTU was 0.13, over 200-fold higher than with no addition, and strikingly high compared with other findings with cultured melanocytes. Cell pellets were brown rather than black in color (not shown). In a separate experiment, melan-a cell pellets were prepared after culture with and without combinations of these factors and TPA (TPA was previously present throughout). Here cells were precultured with 500 μM rather than 300 μM PTU to assist complete depletion of eumelanin, and grown with ASIP etc for 5 days only. The combination of ASIP and high cysteine now gave a tan-yellow cell pellet, or white with continued PTU addition (Figure 2B). Overlap of the exposure to high PTU with ASIP treatment would have prevented the synthesis of any eumelanin in the period before ASIP had taken effect, but reduction of the PTU level was then necessary for any melanin synthesis at all to occur. Supplementation of the precursor cysteine was evidently needed for synthesis of enough pheomelanin to give a yellow color. Pellets of cells grown without TPA were darker than those with TPA, and smaller, reflecting lower cell numbers (slower growth).

### Melanocytes from lethal yellow and recessive yellow mice: ASIP-like effects on cell morphology and growth

For comparison with the effects of ASIP and as further potential pheomelanic melanocytes, we established three independent immortal melanocyte lines from each of two genotypes of yellow mice, by crossing an *Ink4a-Arf* knockout allele into the strain to prevent cell senescence (Sviderskaya et al., 2002). Lines melan-Ay1 to -3 were grown from *A*^*y*^*/a* mice, in which ASIP is expressed ubiquitously from a housekeeping promoter, including in melanocytes, through a genomic rearrangement (Duhl et al., 1994). Lines melan-e1 to -e3 were obtained from recessive yellow (*Mc1r*^*e*^*/ Mc1r*^*e*^) mice, which express a truncated, non-functional MC1R (Robbins et al., 1993). The yellow hair in both genotypes contains almost exclusively pheomelanin by HPLC analysis (Ozeki et al., 1995).

**Figure 3:**
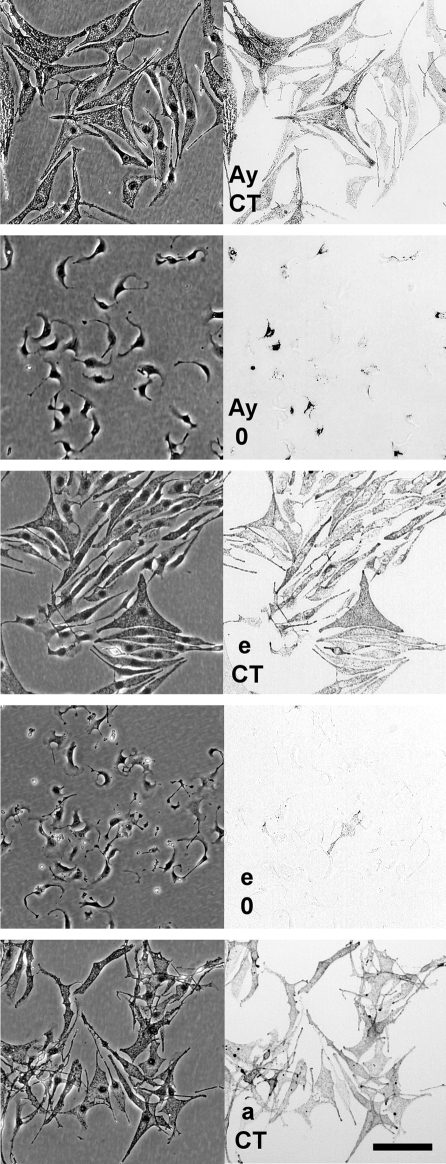
Melanoblast-like appearance of melan-Ay and melan-e cells in the absence of CT. Paired phase-contrast (left) and bright-field (right) images of melan-Ay (Ay) and melan-e (e) cells grown with 20 pM CT (CT) or for several passages without CT (0). Some residual eumelanin is seen in some cells of both genotypes without CT. For reference melan-a cells (a) grown with 20 pM CT are also shown; there is little difference from melan-a cells without CT (Figure 1). Bar = 100 μm.

All lines derived were from *Ink4a-Arf*^−/−^ mice. Melanocytes of both yellow genotypes were difficult to grow, with sparse plating and slow growth in all cultures compared with other genotypes in our experience. Initially the cells were maintained in standard conditions with TPA and cholera toxin (CT, 200 pM) to promote proliferation. Cultures of a given genotype appeared similar. The three melan-e cultures appeared hypersensitive to 200 pM CT, with high dendricity, pigmentation and reduced growth; accordingly CT was varied and stocks were grown with 20 pM CT (which appeared optimal) for a number of passages. Cells in all lines of both genotypes now appeared similar: dark, well-differentiated, large, flat, bipolar or polygonal cells, usually with numerous visible melanosomes, and similar to melan-a cells with 20 pM CT (Figure 3). The cell color by light microscopy and the pellet color (not shown) were black. For further studies a single line of each type was used, melan-Ay1 and melan-e2. Cholera toxin was removed entirely from both lines after passage 10. Growth seemed to be transiently inhibited, then recovered. Both melan-Ay and melan-e cells without CT became small, curly and bipolar, with few dendrites and little pigment, again resembling melanoblasts (Figure 3, compare Figure 1). Melan-Ay cells grew more slowly in growth medium alone than with 20 pM CT or 100 pM NDP-MSH [(4-Nle, 7-D-Phe)-α-melanocyte stimulating hormone, a serum-stable synthetic MSH analog] (doubling times 2.6, 1.6 and 1.5 days respectively, averaged over three passages with duplicate dishes). Likewise, melan-e cells showed doubling times of 2.1 and 1.4 days without and with 20 pM CT. Like melan-a cells with ASIP, some melan-Ay and melan-e cells without CT remained heavily pigmented and dendritic. The change was reversible in both melan-Ay and melan-e cells: when reincubated with CT, they regained the previous melanocytic appearance (melan-e: Figure S1; not shown for melan-Ay).

Melan-e cells responded to very low CT concentrations, becoming pigmented and dendritic with as little as 300 fM CT (Figure S1). These conditions were now utilized to test the specificity of the morphological effects of ASIP. Melan-e cells were grown with or without CT (300 fM) and with or without ASIP (10 nM). Agouti signaling protein had no detectable effect on melan-e cells under either set of conditions (Figure S1). Conversely, melan-a cells with 300 fM CT did respond to ASIP by switching to melanoblast-like morphology as usual (Figure S1), confirming that 10 nM ASIP can counteract 300 fM CT in MC1R-positive cells. Together, these findings show that the induction of melanoblast-like morphology by ASIP is receptor-mediated and specific.

### A carboxy-terminal fragment of ASIP: lack of effect on cell number, shape or pigmentation of melan-a cells

To analyze ASIP signaling further, the ASIP C-terminal analog ASIP-YY was tested. ASIP-YY corresponds to the cysteine-rich domain, ASIP(80–132), with residues 115 and 124 mutated to tyrosine (Y) to improve protein folding (McNulty et al., 2005). This domain of ASIP is sufficient for high-affinity receptor binding in vitro. ASIP-YY displaces NDP-MSH from human melanocortin receptors expressed on HEK-293 human kidney cells, and inhibits αMSH-stimulated cAMP production, demonstrating competitive antagonism (McNulty et al., 2005).

**Figure 4:**
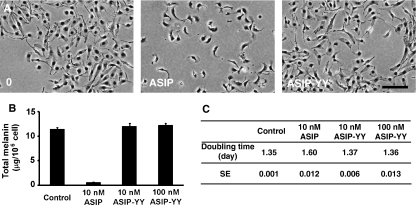
Melanoblast-like shape-change, depigmentation and growth inhibition of melan-a cells by agouti signaling protein (ASIP) but not ASIP-YY. (A) phase-contrast images of melan-a cells grown for 5 days in growth medium (0), with 10 nM ASIP (ASIP) or with 100 nM ASIP-YY (ASIP-YY). Bar = 100 μm. (B) Total melanin content of melan-a cells grown in growth medium only, or with ASIP or ASIP-YY for 14 days. Mean and SEM of triplicate dishes. (C) Cell doubling times of melan-a cells subcultured with no addition, ASIP or ASIP-YY for 30 days. Shown are mean ± SEM of triplicates.

**Figure 5:**
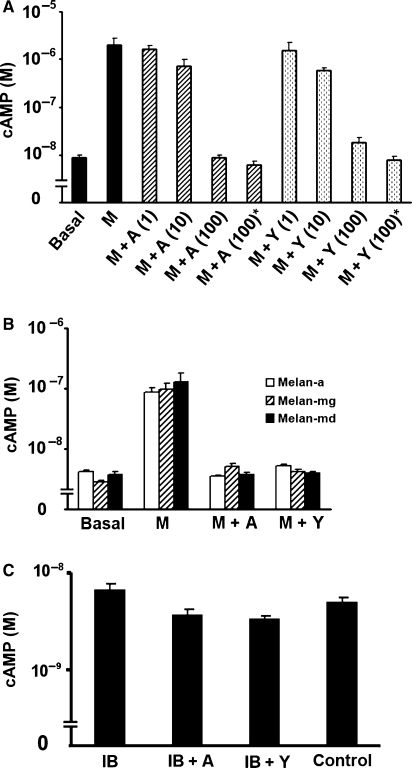
(A) Inhibition of NDP-MSH-induced cAMP rise by both agouti signaling protein (ASIP) and ASIP-YY in melan-a cells. Intracellular cAMP per well was measured in cultures that had been plated and allowed to attach overnight, after stimulation for 30 min with 30 pM NDP-MSH (M), with or without ASIP (A) or ASIP-YY (Y). Figures in parentheses are concentration of ASIP or ASIP-YY in nM. *RPMI1640 medium with 10% FBS was incubated with ASIP or ASIP-YY at 37°C overnight and was used as stimulating buffer, followed by application of NDP-MSH. Shown are mean ± SEM of triplicate wells. (B) Inhibition of NDP-MSH-induced cAMP rise by both ASIP and ASIP-YY in melan-mg and melan-md cells. Assay as above, using 100 nM ASIP (A) or 100 nM ASIP-YY (Y). The reason for the lower apparent stimulation of melan-a cells by NDP-MSH in (B) than in (A) is unknown; it may reflect change in the assay reagents with time. However the relative responses of different genotypes are clear. (C) Reduction of basal cAMP by both ASIP and ASIP-YY. In this experiment only, to increase assay sensitivity, melan-a cells were plated at a higher number of 6 × 10^4^ cells/well, preincubation with isobutylmethylxanthine (IBMX) was for 5 h at 1 μM, and incubation with the stated additives was for 5 h. IBMX alone (1 μM, a low concentration) had no significant effect, whereas both ASIP (A) and ASIP-YY (Y) at 10 nM gave significant reductions in cAMP levels compared with 1 μM IBMX alone (P = 0.013, P = 0.003 respectively). Similar effects were seen under slightly varied conditions (not shown).

Unexpectedly, melan-a cells grown with up to 100 nM ASIP-YY showed no visible difference from control cells (Figure 4A), and no effect on melanin content nor cell growth, although 10 nM ASIP induced the usual shape-change and depigmentation in parallel cultures (Figure 4B, C). To ensure that our ASIP-YY preparation retained its known inhibitory activity, we evaluated its inhibition of MSH-induced cAMP production in melan-a cells. Cells were stimulated with 30 pM NDP-MSH (found to give maximal stimulation in this assay – data not shown), and intracellular cAMP was quantitated by ELISA. NDP-MSH alone induced a more than 200-fold increase in cAMP concentration (Figure 5A, black bars). Neither 1 nM ASIP nor 1 nM ASIP-YY affected this increase, but 10 nM and 100 nM ASIP reduced it by 2/3 and to zero respectively (Figure 5A, striped bars). ASIP-YY did inhibit the cAMP stimulation to a similar extent (Figure 5A, dotted bars). To exclude the possibility that ASIP-YY is rapidly degraded in serum-containing medium, 100 nM ASIP or 100 nM ASIP-YY were incubated with culture medium containing 10% fetal bovine serum (FBS) at 37°C overnight and the mixture was used as stimulating buffer for cAMP measurements. Both ASIP and ASIP-YY still inhibited the MSH-induced cAMP rise completely (Figure 5A, bars with asterisks). Thus, ASIP-YY could inhibit cAMP stimulation as effectively as ASIP. Lastly, as seen in Figure 5C, melanocytes cultured in the absence of added melanocortin showed similar decreases in cAMP level with either ASIP or ASIP-YY. This excluded the possibility that the biological effects were mediated only through an inverse agonist effect of ASIP upon cAMP level, with the effect not being shared by ASIP-YY.

### *Atrn* and *Mgrn1*-null melanocytes: partial biological responses to ASIP but not ASIP-YY

To investigate molecular requirements for the ASIP effects, immortal melanocytes were isolated carrying the null mutations *Atrn*^*mg-3J*^ (melan-mg1 and -2) and *Mgrn1*^*md-nc*^ (melan-md1 and -2). Attractin specifically binds the amino-terminal domain of ASIP, apparently behaving as an accessory receptor for ASIP. *Atrn*^*mg-3J*^ homozygotes are completely resistant to ASIP hair-color effects in vivo (He et al., 2001). The intracellular E3 ubiquitin ligase mahogunin (MGRN1) also seems crucial: *Mgrn1*^*md-nc*^ homozygous mice likewise lack any pigmentary response to ASIP (He et al., 2003).

**Figure 6:**
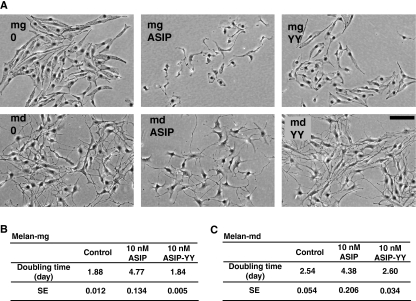
Melanoblastic shape-change and growth inhibition of melan-mg and melan-md cells by agouti signaling protein (ASIP) but not ASIP-YY. Melan-mg cells (mg) and melan-md cells (md) were incubated for 5 days in growth medium alone (0), with 10 nM ASIP (ASIP) or with 10 nM ASIP-YY (YY). Bar = 100 μm. No Cys or phenylthiourea (PTU) present. Cell doubling times of melan-mg cells (B) and melan-md cells (C) were measured in the presence of 10 nM ASIP or 10 nM ASIP-YY for 30 days. Shown are mean ± SEM of triplicate cultures. Retardation of proliferation by ASIP was highly significant for both genotypes by Student’s *t*-test (P < 0.001), while ASIP-YY had no significant effect.

These cultures were from *Ink4a-Arf*^*+/−*^ animals. Again, lines of the same genotype appeared indistinguishable and are not specified here. Growth from both mutant genotypes was initially very slow, attributable to *Ink4a-Arf* hemizygosity, but lines were eventually established. Lines melan-mg2 and -md1 were used for more detailed studies. Both lines retained slower growth than melan-a cells (Figure 6), but this was comparable to growth of the non-mutant cultures from littermate *Ink4a-Arf* heterozygous mice of the same strains: melan-a6 (md control) and melan-a7 (mg control) showed doubling times of 2.4+/−0.10 and 1.9+/− 0.17 days (mean, SEM) respectively. Hence this slow growth was not due to the pigmentary genotype. Melan-mg cells grown without CT had a well-differentiated, flat appearance with numerous black melanosomes (Figure 6, Figure S2). After growth with 10 nM ASIP, they showed a partial response: a somewhat melanoblast-like appearance with variable reduction or clustering of melanosomes (Figure S2), and markedly slower growth, but 10 nM ASIP-YY gave no detectable change in shape or growth rate (Figure 6). Melan-md cells displayed a highly differentiated melanocytic appearance with long, branching dendrites and heavy pigmentation in normal medium without CT (Figure 6, Figure S2). On growth with 10 nM ASIP, there was only a subtle shape change, with shorter and less-branched dendrites, a slight crescentic appearance and cells remaining visibly black-pigmented. However, proliferation was significantly reduced, whereas 10 nM ASIP-YY affected neither morphology nor proliferation (Figure 6).

### Inhibition of MSH-induced cAMP rise by both ASIP and ASIP-YY in melan-mg and melan-md cells

cAMP was assayed in melan-mg and melan-md cells and control melan-a cells after stimulation by NDP-MSH with or without ASIP or ASIP-YY (Figure 5B). Interestingly, both 100 nM ASIP and 100 nM ASIP-YY completely inhibited the MSH-induced cAMP rise in both genotypes, implying that this inhibition requires neither ATRN nor MGRN1.

## Discussion

Here we show that recombinant ASIP induces morphological dedifferentiation of cultured melanocytes to a melanoblast-like shape (reduced cell size and dendricity, acquired crescent shape) and retards growth, as well as strongly inhibiting eumelanogenesis and greatly increasing the pheomelanin to eumelanin ratio, especially in the presence of extra cysteine and PTU. The promotion of pheomelanogenesis by PTU is consistent with the biochemical kinetics favoring cysteinyldopa formation at low tyrosinase activity (Ito and Wakamatsu, 2008). The inhibition of melanogenesis and proliferation by ASIP in melan-a cells were also recently reported by Le Pape et al. (2008), but genetic requirements, receptor-specificity and cysteine effects were not analyzed. There were some minor differences in outcomes, for example they saw a reduction in pheomelanin content with ASIP alone and we did not; these may be attributable to differences in protocol (for example our longer treatment times), in exact culture conditions or in cell passage level. Le Pape et al. (2009) have also now reported a valuable microarray analysis of gene expression changes in melan-a cells grown with ASIP, which supports the concept of partial dedifferentiation and complements the present findings; for example reduced expression of a large number of pigmentary genes including *Mitf* was noted after growth with ASIP.

Several observations in the present study indicate that the morphological dedifferentiation seen with ASIP is a specific effect rather than due to a contaminant. Firstly, the cell shape change was deficient in MGRN1-null melan-md cells and ATRN-null melan-mg cells, and completely absent from MC1R-null melan-e cells grown with a trace of CT. Secondly, the altered cells looked like normal melanoblasts rather than appearing abnormal or unhealthy. We have never observed any other substance to transform melan-a cells in this way; on the other hand ASIP does inhibit forward differentiation of melanoblasts (Aberdam et al., 1998; Sviderskaya et al., 2001). Thirdly, the same melanoblast-like shape was also observed in melan-Ay cells without CT (and without added ASIP), indicating that it could also be induced by endogenous mouse ASIP. Again it was seen in melan-e melanocytes without CT, and it was suppressed in both melan-Ay and melan-e cells by CT.

Surprisingly, these morphological changes and reduced melanin content were not provoked by the ASIP C-terminus (ASIP-YY), although this fully inhibited the stimulation of cAMP by MSH, and reduced the cAMP level in the absence of added melanocortin. Thus, reduced intracellular cAMP is not sufficient for any of the biological effects of ASIP observed here, even though many findings support the sufficiency of increased cAMP for forward melanocytic differentiation [reviews: (Bennett, 1989; Bertolotto et al., 1998)], including the effects reported here of CT, a cAMP agonist.

The growth inhibition by ASIP apparently also required the *N*-terminus of ASIP, as it was not produced by ASIP-YY. However it required neither MGRN1 nor ATRN, as melan-md and -mg cells were also inhibited, if anything more markedly than melan-a cells. This was the only tested biological effect of ASIP not diminished in melan-mg and -md cells. Surprisingly, it appeared independent of the ability to reduce cAMP level, as ASIP-YY affected cAMP but not growth. We cannot entirely discount the possibility that this growth inhibition was partly non-specific, but a counterargument is that slower growth than in wild-type melanocytes also accompanied the melanoblast-like phenotype seen in melan-Ay cells which make endogenous ASIP, and melan-e cells without CT.

Melanocortin receptors and ATRN are the only molecules known to bind ASIP (Abdel-Malek et al., 2001; He et al., 2003). Attractin is required for ASIP effects in the mouse, and was suggested to function to enhance ASIP–MC1R binding (Barsh, 2006). Thus it was surprising that we saw partial morphological and depigmentation effects of ASIP on ATRN-null melanocytes. Perhaps ASIP also has some partial effects in vivo in ATRN-null mice, but they are not sufficient to dilute visibly the black color of hair. Agouti signaling protein (ASIP)’s morphological effects were very deficient in MGRN1-null melanocytes, consistent with the requirement of MGRN1 for ASIP action in mice.

Through which signaling pathway does ASIP/MC1R induce biological effects, if not through reduced cAMP? Each of five melanocortin receptor subtypes couples to adenylate cyclase, generating cAMP and activating PKA (García-Borrón et al., 2005). Other signaling intermediates reported to be activated by MSH/MC1R in melanocytes or melanoma cells include protein kinase C, nitric oxide, p38MAPK and p42/p44MAPK, with the first three reported to stimulate melanogenesis/differentiation, but in each case there was evidence that the effect was dependent on cAMP signaling (Buffey et al., 1992; Englaro et al., 1995; Park et al., 2006; Smalley and Eisen, 2000; Tsatmali et al., 2000). Another candidate pathway is through G_q_ and/or phospholipase C. Coupling to inositol triphosphate (IP_3_) and Ca^2+^signaling (which G_q_ and phospholipase C could mediate) has been reported for a number of human and mouse melanocortin receptors, including MC1R (Eves et al., 2003; García-Borrón et al., 2005; Mountjoy et al., 2001). Recently, a novel high-affinity ligand for MC1R was identified in dogs, namely canine β-defensin 103 (CBD103), encoded at the *K* locus (Candille et al., 2007). Transgenic mice expressing cDNAs for either of two (canine) CBD103 alleles on an *A/A* (agouti) background had a black coat color. Canine and human β-defensins could bind dog, mouse and human MC1Rs, but not ASIP-YY. Interestingly, neither canine cDNA affected cAMP concentration in melan-a melanocytes. CBD103 might act by competitively inhibiting ASIP binding, or might elicit cAMP-independent signaling from the MC1R to promote eumelanogenesis (Candille et al., 2007).

**Figure 7:**
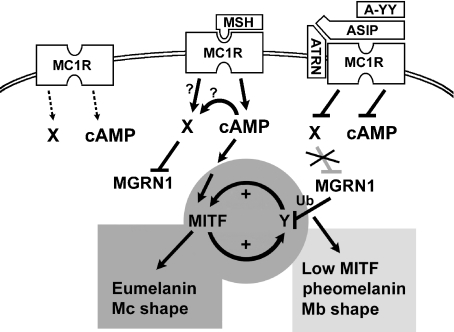
Model to account for responsiveness to cAMP level in activation but not inactivation of eumelanin synthesis. See Discussion for main explanation. (A-YY) ASIP-YY, (Mc) melanocyte, (Mb) melanoblast, (Ub) ubiquitinylation. Melanocortin-1 receptor (MC1R) is shown with some basal activity (left, lost in *e* mutant), to explain why POMC null, *a/a* mice are black, and related data (see text). (This might alternatively be due to BD103 or some unknown signal). Agouti signaling protein (ASIP) would not be required for pheomelanogenesis unless Y has been switched on by the MC1R/cAMP pathway, hence inactive MC1R would give a yellow color even with deficient MGRN1, as seen in *e/e, md/md* mice.

Considering this with our results, we hypothesize dual signaling from MC1R, as follows (Figure 7). Melanocortin-1 receptor can activate both cAMP and another signaling pathway X. Activation of X may be cAMP-dependent or -independent; X may also be the cAMP-independent pathway activated by β-defensins. Agouti signaling protein can antagonize cAMP signaling through its *C*-terminus (as in ASIP-YY) and independently antagonize X signaling through its *N*-terminus and through ATRN and MGRN1, resulting in inhibition of eumelanogenesis, enhancement of pheomelanogenesis (under suitable conditions), and melanoblast-like morphology. To explain the activation of eumelanogenesis by cAMP, yet persistence of eumelanogenesis when the cAMP signal is withdrawn, we suggest a positive feedback loop (Figure 7), likely to involve MITF as MITF mediates melanocytic differentiation. Agouti signaling protein can also downregulate MITF gene expression (Le Pape et al., 2009). The components of this proposed loop remain to be elucidated, but we suggest that MGRN1, a ubiquitin ligase, may break the loop by ubiquitinating and destabilizing one component Y. This would lead to a less-differentiated state with low MITF activity, permissive for pheomelanogenesis.

Full pigment type-switching to give predominantly pheomelanin and a clear yellow color has not been achieved in cultured melanocytes or melanoma cells treated with ASIP (Graham et al., 1997; Sakai et al., 1997), or ASIP and PTU (Le Pape et al., 2008). Likewise, pheomelanin content in melan-a cells was not increased by ASIP alone in this study. With ASIP, cysteine and PTU however, a substantially increased pheomelanin/eumelanin ratio of 0.13 was seen here, although this was not complete pigment type-switching, as the ratio in yellow mouse hair can exceed 100:1 (Ozeki et al., 1995). Moreover, visibly yellow pelletted cells were obtained with a specific combination of intensive PTU pretreatment, ASIP and cysteine, illustrating the sensitivity of the pheomelanin–eumelanin ratio to precise culture history. Further work is needed to produce overt pigment-type switching in culture, but further combinations of the above factors may prove fruitful.

Receptor-specific induction of a melanoblast-like shape from melanocytes by ASIP and a similar effect of MC1R inactivity have not been reported before. The recurrent melanoblast-like morphology in genetically yellow and ASIP-treated melanocytes suggests a possible relationship between dedifferentiation and pheomelanogenesis, supported by the known inhibition of forward melanoblast differentiation by ASIP. *Mc1r*^*e*^*/Mc1r*^*e*^ melanocyte cultures were reported previously, but were grown with dibutyryl cAMP and were described only as pigmented (Costin et al., 2004). Melan-a cells treated with ASIP lose expression of TYRP1, DCT, and SILV/PMEL, all needed to generate normal eumelanosomes but not pheomelanosomes, while retaining low tyrosinase expression (Sakai et al., 1997) which would favor pheomelanogenesis (Ito, 2003). Thus a partial equivalence between melanoblasts and the cells that make pheomelanin seems possible; melanoblasts do express some tyrosinase (Sviderskaya et al., 1995), have low DCT, TYRP1, and MITF (Aberdam et al., 1998), and contain premelanosomes (Hirobe and Takeuchi, 1978). Premelanosomes and pheomelanosomes are evidently not the same, as the latter contain pheomelanin, but the difference may depend on relatively minor factors such as cysteine concentration.

Taken together, our results indicate that ASIP’s biological effects require a cAMP-independent signaling pathway via MC1R and MGRN1 and facilitated by ATRN, which plays a key role in regulating melanogenesis and melanocyte morphology.

## Materials and methods

### Reagents

Tissue culture plastics and FBS were from Gibco Europe (Paisley, UK). Cholera toxin, TPA, PTU (also known as phenylthiocarbamide), NDP-MSH and 3-isobutyl-1-methylxanthine (IBMX) were from Sigma Chemical Co. (Poole, UK). Full length recombinant mouse ASIP was generated using a baculovirus expression system; material used for these studies was purified by cation and anion exchange chromatography as previously described (Ollmann et al., 1998), and was >99% pure. ASIP-YY was synthesized as described (McNulty et al., 2005), was 90–95% pure and has MC1R binding activity equivalent to that of normal ASIP (McNulty et al., 2005). All protein factor stock solutions were prepared in Dulbecco’s phosphate-buffered saline (PBS) lacking CaCl_2_ and MgCl_2_, with bovine serum albumin (1 mg/ml) as carrier, and stored at −70°C. For all experiments involving cysteine addition to culture medium, 100 μM mercaptoethanol was added to enhance cysteine stability.

### Establishment of immortal murine melanocyte lines

Mice were of the C57BL/6J inbred strain and *a/a* at the agouti/*ASIP* locus unless otherwise stated. All experiments complied with local and European legislation concerning vivisection and the experimentation and use of animals for research purposes. Mice with color mutations were crossed to mice carrying an *Ink4a-Arf* deletion allele for one or more generations, to give *Ink4a-Arf*^*−/−*^ or ^+/−^ offspring that yield diploid melanocytes deficient in cell senescence and readily established in culture (Sviderskaya et al., 2002). For melan-Ay-1 to -3, *A*^*y*^*/a, Ink4a-Arf*^*−/−*^ mice were generated from *A*^*y*^*/a* mice kindly provided by FR Gruijl, Leiden University Medical Center, the Netherlands. A dermal fibroblast line of this genotype, fibro-Ay, was also isolated as a potential source of secreted ASIP; not characterized further. For melan-e1 to -e3, *Mc1r*^*e/e*^*, Ink4a-Arf*^*−/−*^ mice were used. For melan-mg1 and -mg2 (*Atrn* null) and melan-md1 and -md2 (*Mgrn1* null), *Atrn*^*mg-3J/mg-3J*^*, Ink4a-Arf*^*+/−*^ mice on a C3H *a/a* background and *Mgrn1*^*md-nc/md-nc*^*, Ink4a-Arf*^*+/−*^ mice on a mixed *a/a* strain background were generated (inbred not available). Neonatal mouse skins were sent to London in chilled culture medium, and used to isolate epidermal melanocytes with the aid of immortal keratinocyte feeder cells as described (Sviderskaya et al., 1997, 2002), using RPMI1640 medium (Sigma) containing 10% FBS, 200 nM TPA, and 200 pM CT, gassed with CO_2_ (10% v/v). Routine subculture was as described (Sviderskaya et al., 1997). Littermate wild-type lines were also established as potential strain controls (melan-a5 and -a6 from the *Mgrn1*^*md-nc*^ stock and melan-a7 from the *Atrn*^*mg-3J*^ stock); these resembled other black melanocyte lines. After passage 10, CT was removed from all cell lines without cessation of proliferation. Melan-a cells (Bennett et al., 1987) were grown in the same medium (no CT). Melan-a cells are functionally *Ink4a-Arf* null, expressing neither p16^INK4A^ nor ARF proteins (Sviderskaya et al., 2002).

### Melanin and cell proliferation assays

Lyophilized samples of 0.5–1.0 × 10^6^ melanocytes were processed for chemical analyses to detect the specific degradation product of eumelanin, pyrrole-2,3,5-tricarboxylic acid (PTCA), after permanganate oxidation (Ito and Fujita, 1985; Ito and Wakamatsu, 1994) and the specific degradation product of pheomelanin, 4-amino-3-hydroxyphenylalanine (4-AHP), after hydriodic acid hydrolysis (Ito and Fujita, 1985; Wakamatsu et al., 2002). Both products were quantified by HPLC. The amounts of eumelanin and pheomelanin were obtained by multiplying the amounts of PTCA and 4-AHP by conversion factors of 50 and nine respectively. For measurement of total melanin (Friedmann et al., 1990), cell pellets were resuspended in 100 μl NaOH (1 M) and diluted with 400 μl water. The OD_472_ was measured and converted to melanin content via a standard curve using synthetic melanin (Sigma). This was normalized to cell number.

To calculate cell doubling times, triplicate cultures were plated at the specified density on 3-cm dishes. When cells were nearly confluent, they were detached with trypsin-EDTA, counted in triplicate by hemocytometer, and replated at a recorded density. A growth curve was drawn from the relative population increase. Cell doubling time over 30 days’ growth was calculated from the curve.

### cAMP assays

Cellular cAMP concentrations were measured using the HitHunter™ cAMP assay kit (Amersham Biosciences, UK), according to the manufacturer’s instructions with slight modifications. Cells were plated into 96-well plates at 2 × 10^4^ cells/well, 100 μl medium/well and incubated at 37°C in normal culture conditions overnight. Medium was gently aspirated. Then, unless specified, cells were incubated in serum-free RPMI1640 with BSA (1 mg/ml) and 0, 10 or 100 nM ASIP at 37°C in a humidified atmosphere of 10% CO_2_ for 30 min. To assess the effect of incubation of ASIP in medium, RPMI1640 containing 10% FBS and 100 nM ASIP was incubated under the same conditions for 12 h and this medium was applied in place of the medium with fresh ASIP. For cell stimulation, NDP-MSH (30 pM) and IBMX (1 mM) were added directly to the ASIP-containing medium and cells were incubated at 37°C for 30 min. The cells were lysed and processed according to the manufacturer’s protocol. One hour after the last reaction step, the luminescence of each well was measured with a Synergy™ HT Multi-Mode microplate reader equipped with KC4 for Windows data-reduction software (Bio-Tek Instruments, VT, USA). The cAMP concentration in each sample was calculated by comparing relative luminescence of the sample with that of a cAMP standard.

## Supporting information

**Figure S1.** Lack of morphological effect of ASIP on melan-e cells.

**Figure S2.** Reduction of visible pigmentation in melan-mg and melan-md cells grown with ASIP but not ASIP-YY.
