# Supplementary figures and images for: Agouti protein, mahogunin, and attractin in pheomelanogenesis and melanoblast-like alteration of melanocytes: a cAMP-independent pathway
Source: Pigment Cell Melanoma Res. 2009 Oct;22(5):623–34. doi: 10.1111/j.1755-148X.2009.00582.x (PMC2784899; doi:10.1111/j.1755-148X.2009.00582.x)

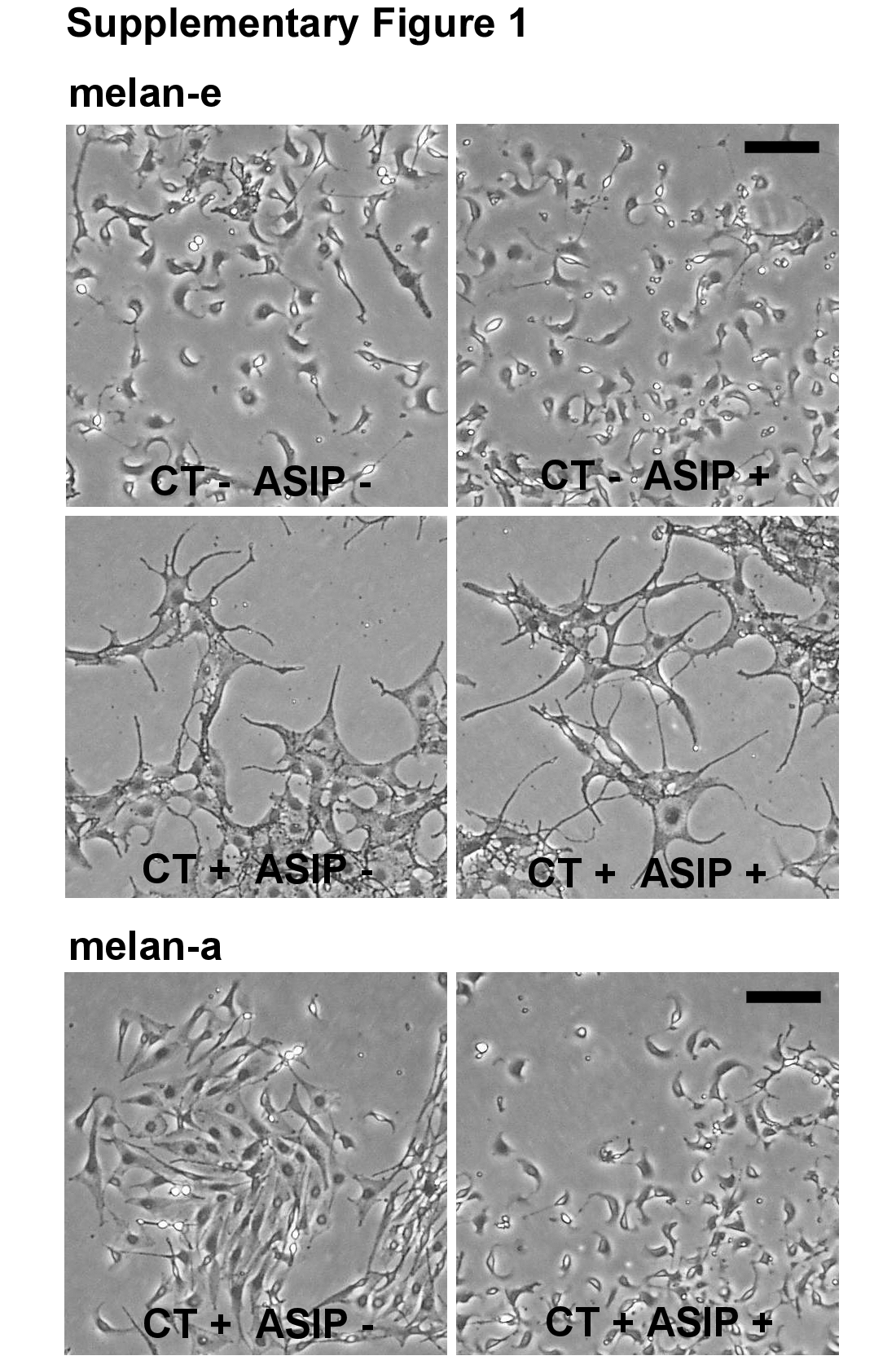

Supplement: Supplementary file 1 [file pcr0022-0623-SD1.tif]

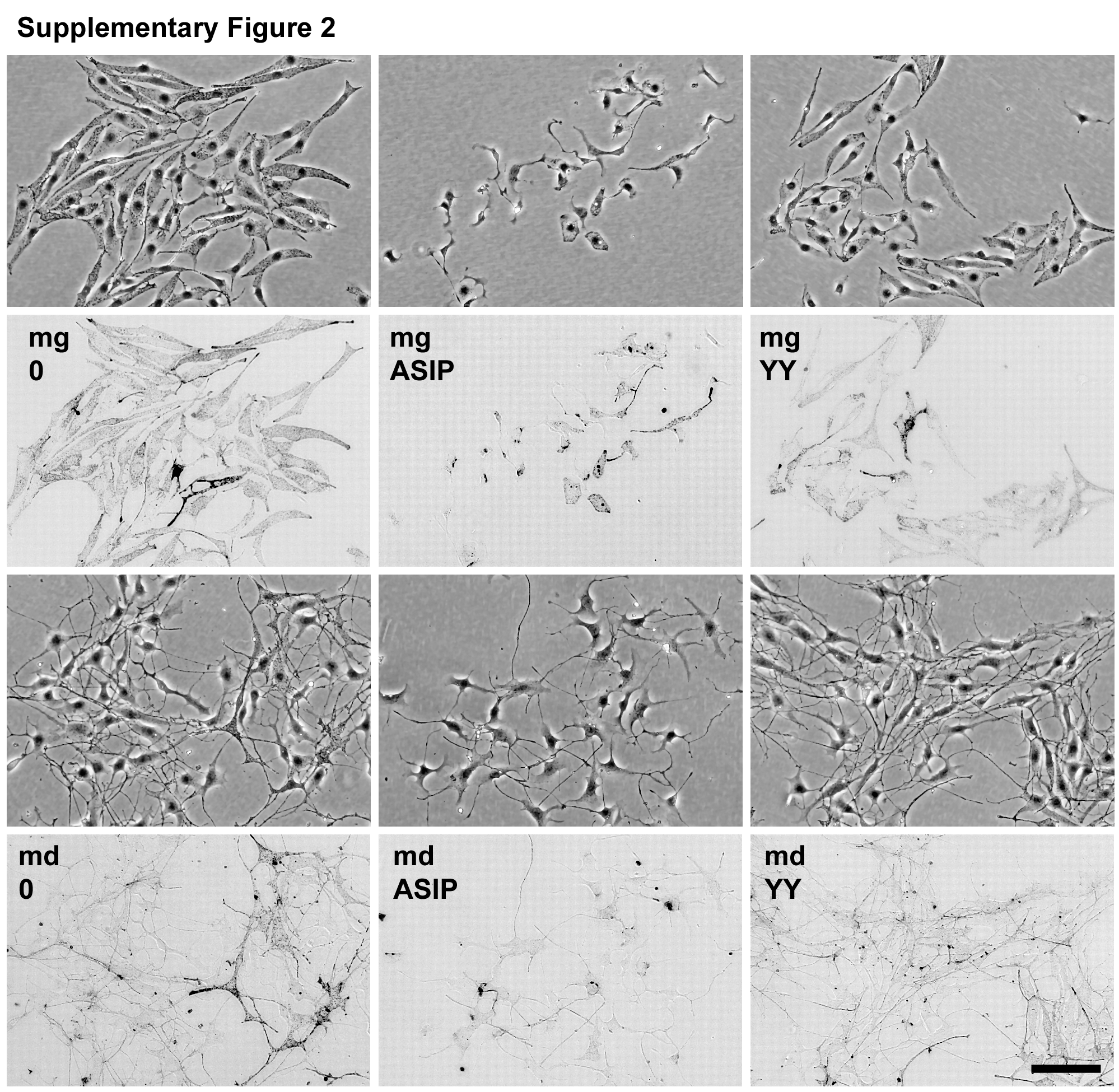

Supplement: Supplementary file 2 [file pcr0022-0623-SD2.tif]
